# Experimental Studies and Application of Fiber-Reinforced Polymers (FRPs) in Civil Infrastructure Systems: A State-of-the-Art Review

**DOI:** 10.3390/polym16020250

**Published:** 2024-01-16

**Authors:** Jorge Albuja-Sánchez, Andreina Damián-Chalán, Daniela Escobar

**Affiliations:** 1 Faculty of Engineering, Laboratory of Materials Resistance, Soil Mechanics, Pavements and Geotechnics, Pontificia Universidad Católica del Ecuador (PUCE), Quito 170143, Ecuador; adamian563@puce.edu.ec (A.D.-C.); descobar239@puce.edu.ec (D.E.); 2 International Faculty of Innovation PUCE-Icam, Pontificia Universidad Católica del Ecuador (PUCE), Quito 170143, Ecuador

**Keywords:** polymer composites, fiber-reinforced polymers (FRPs), civil infrastructure

## Abstract

The application of FRPs in civil infrastructure has increased, particularly in the last 20 years. FRPs have gained importance because of their resistance to harsh environments, high strength-to-weight ratio, and good corrosion resistance, and they are faster and easier to apply than other traditional methods. The rehabilitation of structures is the main area in which FRPs have been developed, because they have allowed for compliance with architectural restraints in historic structures. This review is a compilation of the research conducted on the laboratory and field applications of FRPs, highlighting the different applied methods, installation difficulties, and failure modes of FRPs. Moreover, this review compares studies on the types of fibers such as CFRPs, GFRPs, and AFRPs, and their effects would affect the mechanical properties of civil infrastructure and the durability characteristics of civil infrastructure in challenging environmental conditions. In addition, this review focuses on the modification of the mechanical properties of structural elements using different methods of installing FRPs, including externally bonded reinforcement (EBR), and their main problem: debonding failure before the ultimate load.

## 1. Introduction

The use of fiber-reinforced polymers (FRPs) in the construction industry has gained importance because of their resistance to harsh environments, high strength-to-weight ratios, and good corrosion resistance. They are faster and easier to apply than traditional methods [1,2,3].

FRPs have been applied since WWII, but the use of fibers in the construction industry has been developed in two areas: (i) rehabilitation structures (reparation, strengthening, or retrofitting) and (ii) new constructions using FRPs or new composite FRP/concrete systems [4,5,6].

Since 2006, fibers (polypropylene, carbon, polyethylene, polyester, cellulose, steel, etc.) have been extensively studied as reinforcing materials for concrete, and it was found that the use of fibers enhances the compressive strength, durability, and permeability [3]. Some investigations have shown that the length, diameter, and concentration can modify the mechanical properties.

The influence of different lengths, diameters, and concentrations of carbon nanotube-reinforced cementitious materials on the mechanical properties showed that each size has unique characteristics and mechanisms affecting the properties of concrete structures; for example, small fiber diameters benefit the compressive strength but adversely affect the flexural strength [7].

The first research on FRPs was conducted in the late 1990s and in the early 2000s [4]. The initial results of some investigations and reviews have shown that FRPs are effective for repairing structures. Nevertheless, the rehabilitation of structures requires an appropriate design system, in which the principal objective is the correct use of the material [3,4].

According to Kraków’s Charter (2000), which prioritizes the conservation of patrimonial buildings, the preservation of structures can be realized through different types of interventions, such as the repair, renovation, or rehabilitation of structures. This can justify the application of FRPs in historical buildings as the best rehabilitation method [8]. The advantages of FRPs are that they permit compliance with architectural restraints, the mass of structural elements does not increase, and buildings can gain seismic resistance [9]. Additionally, by increasing the tensile force, the application of FRPs may reduce the quantity of reinforcing bars required for the flexural capacity of composite beam-to-column connections [10].

For construction, an FRP is employed to improve the mechanical properties of reinforced concrete (RC) in terms of flexural and shear resistance [2], as well as resistance to corrosion and other chemical attacks, such as chloride and sulfate [1,5].

FRPs can be classified according to (i) the composition of their fibers and (ii) their use. The most commonly used fibers are (i) natural FRPs (NFRPs) and (ii) synthetic fibers (FRPs) [11]. Scientists are currently developing methods, techniques, and materials that can reduce the environmental impact of construction and be more adaptive to circular economy announcements [12].

An FRP is considered an NFRP when the polymer matrix is a biodegradable polymer reinforced with natural fibers; these last materials can be abaca, bamboo, coconut, or other fibers. The most remarkable characteristic of an NFRP is its low impact on the ecosystem, and its manufacturing process is significantly less harmful to the environment than that of synthetic fibers; however, its disadvantages include poor durability when exposed to water or alkaline compositions and lower strength [11].

An FRP has two constituents: the first is synthetic fibers, whose most common uses are (i) carbon (CFRP), (ii) glass (GFRP), and (iii) aramid (AFRP) [6]. In contrast, the “matrix”, whose function is to keep fibers joined by an epoxy resin system [13,14], can be made with different types of materials, such as metals, ceramics, and polymers. A CFRP is the most commonly used polymer because it is easy to manufacture and relatively less expensive than other polymers [6].

The rehabilitation of structures is the main area in which FRPs have been developed, and several methods exist depending on their application [15]. The methods used for structural reinforcement include (i) near-surface mounted (NSM), (ii) externally bonded reinforcement (EBR), and (iii) externally bonded reinforcement on grooves (EBROG).

**Figure 1:**
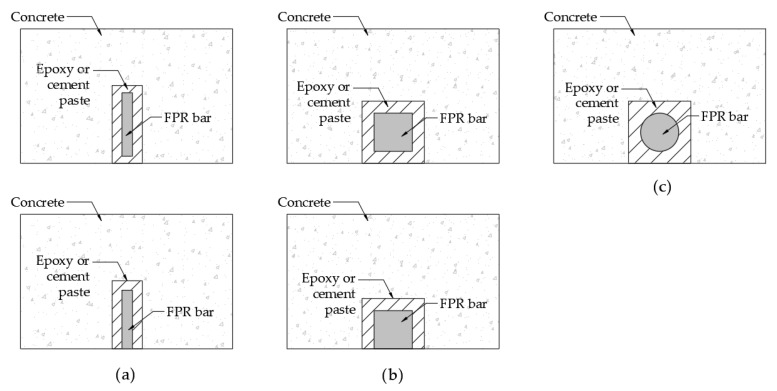
NSM systems with different bar shapes for FRP: (**a**) NSM with plate-shaped bar, (**b**) NSM with rectangular bar, and (**c**) NSM with round bar.

**Figure 2:**
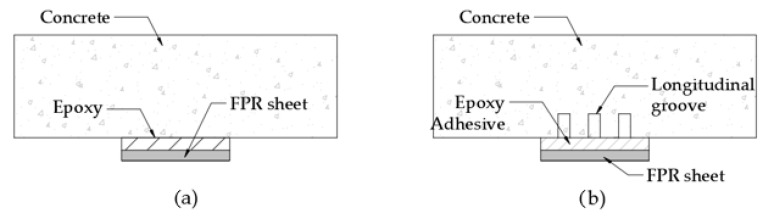
Methods used for structural reinforcement: (**a**) EBR system and (**b**) EBROG system.

Figure 1 shows the different methods used in the NSM method and the types of bars that could be used for reinforcement. Meanwhile, Figure 2 shows the application of the EBR and EBROG methods.

FRPs in new construction are generally used in construction systems. In general, they are coupled with other materials such as concrete because they do not have sufficient stiffness to be independent structures [16].

This review is a compilation of the research conducted on the laboratory and field applications of FRPs and the different applied methods.

## 2. Methodology

The research process consisted of recollecting papers on experimental study cases and the application of FRPs in full-scale or constructed structures. The papers were selected based on (i) publication year, (ii) proposal of the investigation, and (iii) method of obtaining results, the parameters of which are detailed in Table 1.

**Figure 3:**
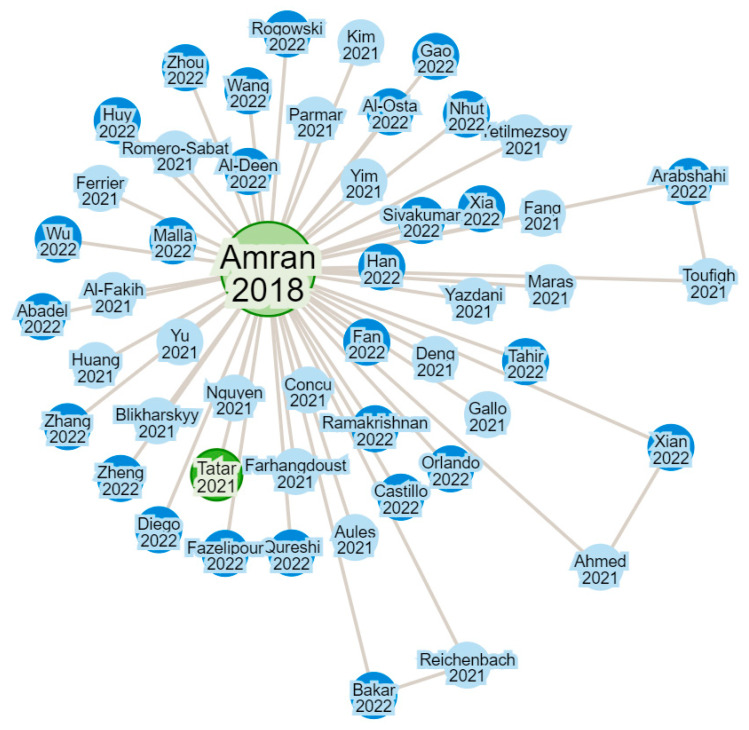
Recollected FRP articles network in the webpage of Research Rabbit.

With these parameters, the search for investigations began, and the tool used to obtain papers was “Research Rabbit”. Using this tool, we created a multitude of collections based on the topics and sub-topics of this review. The base papers to search for were review papers that were cited in another recently published experimental study case paper,

Once the research articles that form the basis for the collection of information were selected, the search tool showed a connection of research without limitations on topics and year of publication, as shown in Figure 3; therefore, the articles quoted in the base article were obtained. Considering the parameters described in Table 1, the items that formed the basis of the review were selected.

It is important to mention that the articles obtained through the tool were published until 2022; therefore, a different methodology was applied for recent studies.

For recent research, that is, from 2023 onwards, a traditional method was used, in which the tools of Scopus and Science Direct were used: “FRP + experimental” was placed in the search bar; in addition, the year filter in which it is delimited, from 2019 to 2024, was occupied.

## 3. Research Progress

### 3.1. Mechanical Properties of FRPs

FRPs were originally used in the automotive, marine, oil and gas, and aerospace industries because of their high strength, low weight, and high modulus. However, in the civil industry, it is more difficult to consider them as elements and/or system constructions because of their high manufacturing costs, and they are more complex to install [6].

The large number of fibers in the market for structural reinforcement has enabled various cost–benefit solutions, as well as different responses to several objectives, to be obtained. Many investigations, whose aim was to study the mechanical properties, affirm that FRPs have a high modulus of elasticity, high tensile strength, good fatigue resistance, high dimensional stability, low coefficient of thermal expansion, and low abrasion [5].

Sreekumar Kavitha et al. [5] compared the laminates of CFRP and GFRP applied on concrete cylinders, which received a 28-day curing period, and found that the CFRP laminates obtained a flexural strength of 162.30 MPa; on the contrary, BFRP reached 114.50 MPa of flexural strength, and the ductility enhanced. Consequently, the results indicated that CFRP and BFRP enhance the stiffness and bond shear streets. CFRP laminates exhibited superior behavior in resisting stress compared with BFRP laminates. In addition, the authors found that the specimen failed owing to cracking, whereas the FRP had a vertical crack, similar to the conditions of studies with FRP laminate confinement.

Numerous studies have demonstrated that concrete wrapped with an FRP jacket can increase its load-bearing capacity for compression and the ultimate axial strain. Therefore, many studies introduced FRP jackets to prevent the lateral deformation of concrete [17].

**Figure 4:**
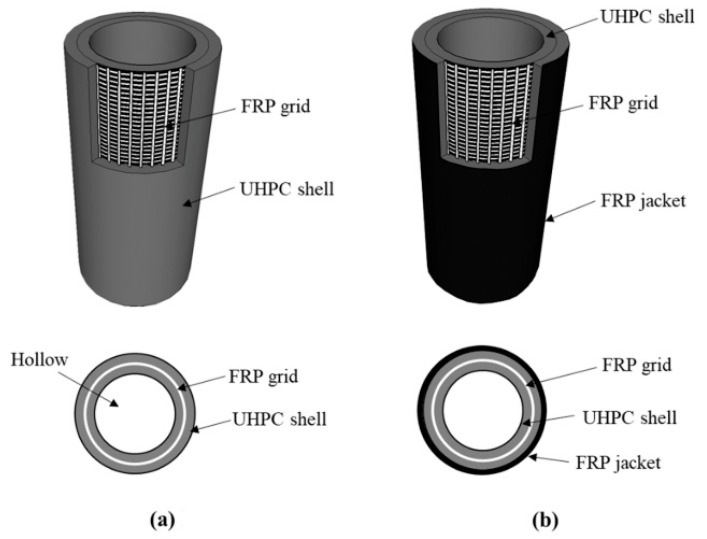
Configurations of the FRP grid and CFRP jacket proposed by Liao et al. [18], (**a**) FRP grid-UHPC pipe; (**b**) FRP grid-UHPC pipe (with external FRP).

Liao et al. [18] proposed ultra-high-performance concrete (UHPC) pipes internally reinforced with FRP grids and CFRP jacketing, as shown in Figure 4.

The objective of this study was to compare them with steel pipes and normal-strength concrete pipes. The authors mentioned that the CFRP jacket could be adopted for protection against corrosion, was nerveless, and obtained better flexural performance than the FRP grip. Therefore, the CFRP jacket can assist in avoiding pipe-side section separation failures [18]. Furthermore, the specimens presented a separation between the FRP grip and UHPC that could be resolved by increasing the pipe thickness or applying CFRP jacketing. The failure mode of the FRP jacket is characterized by fiber rupture, owing to the hoop tension caused by the lateral deformation of the concrete [17].

**Figure 5:**
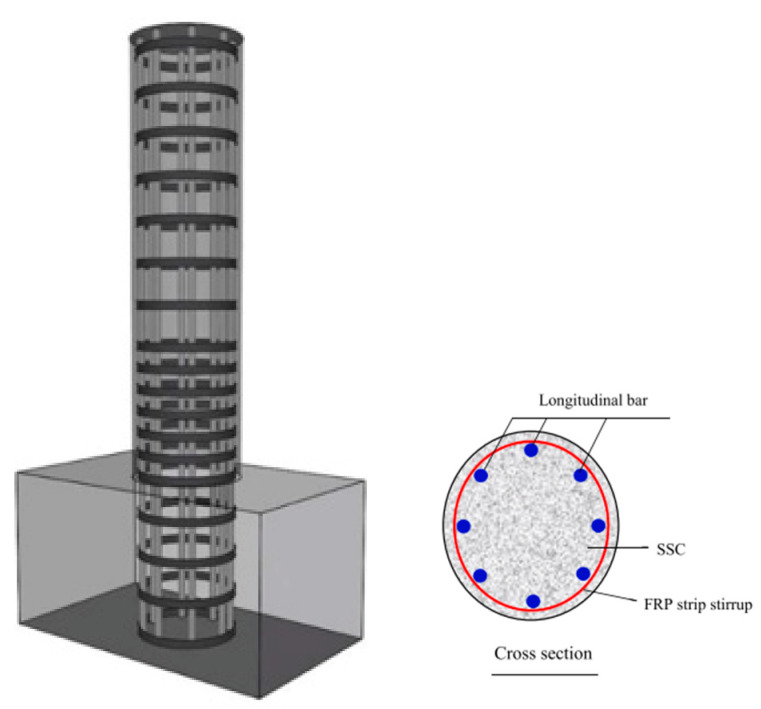
Application of FRP strips in compressive casting sea sand concrete columns [19].

Some investigations have demonstrated novel methods for the construction and improvement of the mechanical properties of structural elements. Yuan et al. [19] developed a study on the axial compressive behavior of sea sand concrete columns confined with CFRP by employing the compression casting method, in which, after curing the cylinders, the CFRP strips are wrapped around the cylinders, as shown in Figure 5.

The results showed that the compression casting method enhanced the ultimate axial strain despite the rapid development of cracks, and failure more suddenly occurred for the compression-cast SSC columns. In addition, FRP reduces its efficiency because of the application of the compression casting process.

**Figure 6:**
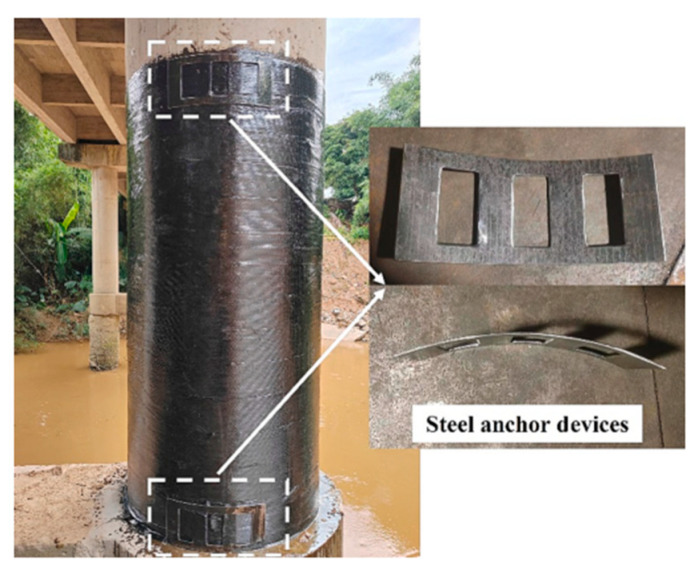
The bridge pier after strengthening [17].

Studies have proposed that an FRP jacket be formed by wrapping a continuous FRP strip at a small angle with respect to the failure angle. Chen et al. [17] applied this technique to circular concrete columns with GFRP strips. In contrast to Liao et al. [18], the failure mode was the rupture of an FRP strip with an explosive sound. The FRP rupture was located near the mid-height region; consequently, the concrete lost its confinement. Based on the results, the study aimed to rehabilitate the piers of a bridge located in Yunfu City, China, using FRP strips, because the bridge became unsafe to operate and presented circumferential cracks on the surface of one of its piers. The wrapping angle was 2.55°. Both ends were strengthened using an additional CFRP strip anchored by a special steel anchor, as shown in Figure 6.

Consequently, it has been proven that the confinement effect of FRP sheets enhances tensile strength. Koushfar et al. [20] experimented with the grouted slice sleeve connection (GSSC), using sheet materials of CFRP and GFRP, and found that the GSSC wrapped in FRP achieved greater tensile strengths than that not using confinement. Moreover, the number of FRP layers could improve the confinement of the grouted splices; however, the rate of increase in tensile strength was not significant.

**Figure 7:**
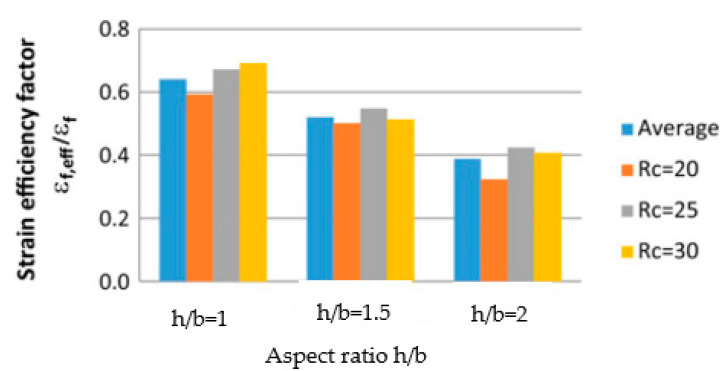
Effect of aspect ratio on strain efficiency factor. (Rc: corner radius, mm).

De Diego et al. [21] affirmed that FRP jacketing is an effective technique to increase strength and strain capacity. Their investigation consisted of analyzing the behavior of FRP-confined concrete under an axial load. The strain efficiency factor decreased with an increase in the cross-sectional aspect ratio, as shown in Figure 7.

Kalyani et al. [22] evaluated the combination of GFRP and AFRP on sheets and reinforcement beams with the EBR method. RC beams were tested using a four-point bending test with a loading frame that was applied to the beam specimens using a hydraulic actuator. The results showed that the flexural capacity of the beams increased by 102.63–202.63%. The authors discussed the possibility of combining various types of fibers to improve their mechanical properties [23]. Wang et al. [24] demonstrated that combining fibers increases the load-bearing capacity and elastic modulus. Their study found that the combination of GFRP, BFRP, and flax FRP resulted in an improvement of 100–110.7% in the load capacity and an increase in the elastic modulus of 11.4–13.9%.

Liao et al. [18], Chen et al. [25], Kalyani et al. [22], and others experimented with reinforcement concrete (RC) because steel is the traditional method for improving the mechanical properties of RC. Prakash et al. [26] compared steel reinforcement with GFRP bars. Their study investigated the behavior of short circular RC columns in terms of compressive strength and ductility. Three specimens each with 100% steel reinforcement (SS) and three specimens that had 50% steel and 50% GFRP reinforcement (SSG) were tested. The results showed that the SSG column had an 11.4% higher axial compression capacity than the SS column. Moreover, the weights of the SSG columns were 1.5% lower than those of the SS columns. Additionally, the authors mentioned that GFRP could be a suitable alternative to steel bars in marine environments.

A combined study by Liao et al. [18] and Prakash et al. [26] reduced the use of steel or FRP bars for reinforcement. The combination of bars and laminates/sheets could increase the ductility; consequently, the cracks could be reduced, and the concrete structure could aim for a higher load capacity.

#### 3.1.1. Harsh Environments’ Resistance to FRPs

FRPs offer high strength-to-weight ratios and excellent corrosion resistance; however, field exposure has not been considered in some studies. Many studies investigated the effects of environmental conditions on the mechanical properties [27] and affirmed that harsh conditions can degrade the mechanical properties of FRP, although some types of FRPs, such as AFRP, are better for use in harsh conditions.

AFRP was demonstrated to exhibit better resistance in marine environments than CFRP and GFRP [28]. AFRPs are frequently used in tunnels, bridges, and road structures owing to their thermal stability, impact resistance, and good insulation properties [1]. The high resistance of AFRPs in marine environments was confirmed by Toufigh et al. [27]. It was found that the bond strength increased after the specimens were brought under acidic environments, and the reduction in the AFRP and CFRP specimens was less than 10% compared to GFRP, which was 18%.

A similar investigation developed by Wang et al. [28] analyzed the effects of Wetting/Drying Cycling (WDC) by adding a salt solution (NaCl) such as seawater. The research methodology involved conducting a series of experiments in which FRP strips were bonded to concrete specimens. The specimens were subjected to wet–dry cycling using a salt solution, simulating the environmental conditions that FRP–concrete composites may encounter in real-world applications such as coastal structures or transportation infrastructure in winter climates.

Various parameters, including bond strength, debonding mode, and failure patterns, were assessed to analyze the effect of salt solution wet–dry cycling on the FRP–concrete interface. Although the objective of this research was to evaluate the degradation of bonding between FRP and concrete, this research also mentioned that the bond behavior of basalt fiber-reinforced polymer (BFRP)–concrete was weaker than that of CFRP–concrete. This difference can be attributed to the use of CFRP, which has a higher tensile strength and modulus of elasticity (T = 4132 MPa; EM = 231 GPa [28]) than BFRP (T = 2100 MPa; EM = 91 GPa [28]).

BFRP has a relatively low price, improved fire resistance, and better resistance to chemical environments than GFRP does. These properties can be attributed to basalts originating from volcanic magma. However, GFRP is less brittle than the other FRPs [29]. Yeboah et al. [29] reported the results of the ultimate load for timber beams strengthened with NSM GFRP and BFRP bars, which showed an increase of 33–67% and 62–69%, respectively.

A combination of fibers was suggested by Kalyani et al. [23]. The purpose of this study was to evaluate the behavior of CFRP and GFRP under harsh temperature conditions (100–500 °C). The experimental results showed that FRP has a lower temperature resistance than stainless steel wire mesh because the specimens disintegrate beyond 200 °C and cannot resist any tensile force. In addition, the strength of the RC beams increased by 81%, owing to the increase in FRP thickness.

As we can see in Section 3.3.1, the debonding failure (DF) is the main problem of EBR [30], as it can increase under aggressive environmental conditions, and the real bond strength can be reduced by 25% [28]. Harsh conditions, such as temperature, acidic environments, and/or WDC, increase the debonding failure. The effect of WDC exposure was investigated by Li et al. [31], who evaluated RC beams with prestressed CFRP under 90 days of WDC conditions. The beams were tested in a flexural study and monitored using fiber Bragg grating sensors. The results indicated that three of the five specimens had a debonding failure; however, the specimens with prestressed CFRP under 90 d of WDC presented a better cracking load and ultimate load than the unreinforced RC beam. In addition, the WDC exposure deteriorated the bonding between the CFRP and concrete, and the cracking loads of the specimens with 40% prestress decreased by 10.79% after 90 days and 15.12% after 180 days.

However, in this study, WDC exposure was reached in 180 days fewer than in the study by Wang et al. [24], while both studies reached the same conclusion: WDC exposure significantly reduces the stiffness and bond shear streets, with reductions of 50.1% and 39.7%, respectively, which permits the definition of CFRP fibers to be the best for use in marine exposure environments [21].

Zhang et al. [32] investigated the behavior of RC beams with AFRP exposed to saltwater using high-strength and normal-strength concrete. Similar to other investigations, the DF also affirmed that the failure modes of AFRP did not change with the immersion time. Although the cracking load increased with the immersion time, it did not significantly affect the load-carrying capacity.

A recent review of the effects of FRP underwater was conducted by Yu et al. [1], where the bonding resistance decreased by approximately 22.6–34.2% for the pull-off bond test and 14.8–20.0% for the shear strength test. The research concluded that water immersion and salt erosion are the two main reasons for the deterioration of FRP–concrete bond behavior.

To adapt to the conditions at a site, it is necessary to adapt to the materials present in the environment. Sea sand and coral concrete have poor mechanical performance; however, the increasing construction activities on islands make FRPs a solution, owing to their good corrosion resistance. Zhou et al. [33] added fibers as reinforcement in coral concrete and obtained a reduction in the cracks in the concrete, which also changed the bond–slip curve. Similarly, Al-Fakih et al. [34] conducted a study on CFRP plates incorporated with sea sand concrete and bonded to RC beams incorporating sea sand bonded with CFRP plates. The results were the DF for all the specimens; however, the CFRP plate increased the stiffness and cracking performance.

For example, Liu et al. [35] demonstrated that the incorporation of CFRP plates ameliorates the bearing capacity and stiffness capacity, and RC beams with sea sand concrete presented better results than RC beams alone, although they had worse results than RC beams bonded with CFRP plates. However, each RC beam experienced a DF.

This could be considered a harsh condition because fire damage is a severe threat to the construction of reinforced concrete. The exposure of concrete to fire can affect its stress–strain characteristics and durability. Abadel et al. [30] evaluated the enforcement grade of GFRP by applying after-fire effects. The results indicated that GFRP increased the strength, strain at peak strength, and post-peak behavior for different types of fibers utilized at various heating temperatures and cooling regimes. However, the authors indicated that it is necessary to carefully analyze the application of FRP to repair fire damage. FRP should only be considered when the damage is insignificant.

#### 3.1.2. FRP Behavior under Seismic Loads

However, several FRP structures are in operation, and Siwowski et al. [3] suggested that it is necessary to conduct further research on and demonstrations in large-scale projects to be more competitive with the traditional materials used in construction.

This last statement was reached by Shen et al. [36], who seismically evaluated the behavior of corroded RC shear walls with BFRP. The RC shear walls included the foundation and walls with various corrosion rates that were repaired using BFRP jackets. BFRP debonding, the local buckling of the longitudinal reinforcement, and bottom concrete crushing were the failures. However, it was possible to recover the shear strength capacity using BFRP jackets.

Moreover, the recovery of the original displacement ductility ratio and drift was possible in RC shear walls with 3% and 9% corrosion. Nevertheless, a wall with a corrosion rate of 15% cannot be recovered, as expected, owing to flexural cracks [36].

Similarly, Li et al. [37] evaluated BFRP for reinforced concrete joint beam–columns with different corrosion levels (0.3%) after seismic effects. The results indicated improvements in the ultimate displacements, stiffness, and total cumulative energy dissipation of 14.6%, 16.7%, and 23.3%, respectively. The configuration and distribution of FRP considered the recommendations of the anchorage and position of FRP in the same way as Mahdavipour et al. [31]. Although Gao et al. [38] did not investigate the effects of corrosion under seismic loads, it was found that GFRP could improve the corrosion damage produced by freeze–thaw cycles. Moreover, GFRP changed the mode of failure from brittle shear failure to bending shear failure.

Del Rey Castillo et al. [39] talked about the effectiveness of FRP strengthening in enhancing the seismic performance of RC columns. This study investigated the effectiveness of FRP sheets and anchors in RC columns. This study concluded that the columns exhibited similar behavior, with the first elastic state followed by an inelastic state at the peak load. The lateral load decreased, and the lateral displacement increased until all the longitudinal FRP materials ruptured. One column was applied to the bond-breaking layer between the FRP and concrete. This layer, a novel method, can enhance the ductility capacity of columns.

As mentioned by Siwowski et al. [3], in large-scale studies, Gattesco et al. [40] investigated the effectiveness of applying composite reinforced mortar (CMR) to the external faces of masonry buildings. The CMR consisted of a mortar coating reinforced with a GFRP mesh and injected steel transverse connectors. The building used was a structure designed to represent a historic rural masonry house typical of Italy and Slovenia, which was pre-damaged by a seismic event to simulate a repair intervention.

**Figure 8:**
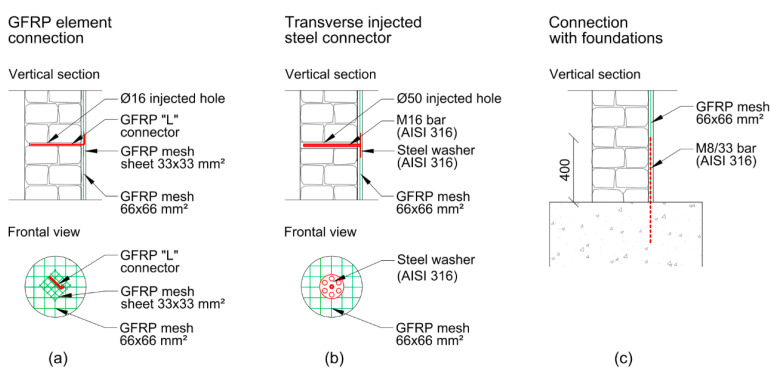
Connection to wall: (**a**) GFRP elements, (**b**) steel connectors, and (**c**) connection to foundation [40].

For the unreinforced masonry building, the resistance increased by 2.4 times, the displacement capacity by four times, and the total dissipated energy by approximately 7.2 times. In addition, the experiment demonstrated that it was necessary to have a connection between the coating and foundation, and steel connectors were used to prevent the separation of the wall [40], as shown in Figure 8.

The analysis of the strengthening of timber structural elements has increased in recent years owing to existing historical structures. Yeboah et al. [29] performed an investigation of the behavior of a structural timber beam reinforcement with NSM BFRP and GFRP bars. A series of 20 beams was tested under displacement control, and the results showed that the BFRP and GFRP increased by 60% of the ultimate load; however, the displacement at failure increased by 34% compared with the control beams. The failure mode was the brittle tensile failure of the timber in the tensile zone.

A [41] evaluated the behavior of on-timber columns under a lateral cyclic loading test with mounted steel bars wrapped with CFRP strips and found that the bearing capacity could improve by 32.3–60.1% and that the deformation performance could be reduced by 67.9–89.4%.

Strip stirrups were used to confine the concrete elements. Wu et al. [42] investigated the shear performance of RC beams with CFRP strip stirrups at two different positions: tested under a static load and fatigue loading. The results indicated that CFRP strip stirrups improved the shear bearing capacity compared with traditional steel stirrups, and CFRP changed the failure mode owing to its ability to control cracks and deflections under fatigue loads.

### 3.2. Novel Types of Fibers Use for Reinforce

Another new fiber is used to improve the dynamic properties of concrete structures. Large rupture strain FRPs (LRS FRPs) are novel fiber composite materials that have emerged in the last decade. Generally, they are composed of polyethylene naphthalene or polyethylene terephthalate (PET). LRS FRPs enhance the ductility, impact resistance duration, and damage degree of concrete specimens [43,44]. Shi et al. [44] experimented with a shear wall under the pendulum impact test. The results indicated that LFRP can significantly improve the impact-bearing capacity, deformation capacity, ductility, and energy dissipation [43].

A comparison between CFRP and LRS FRP was performed by Mei et al. [43]. LRS FRP is superior to CFRP in seismic retrofitting to CFRP due to its deformation capacity, which exceeds 5% of that of other FRPs. Moreover, the study demonstrated that CFRP-strengthened columns under a high axial load experienced explosive failure, whereas LRS FRP experienced progressive failure.

Synthetic fibers have been tested in diverse studies, and it has been proven that this type of FRP improves mechanical properties, such as flexural and shear resistance. However, Chen et al. [11] studied the effect of using flax and jute as an NFRP using the EBR method with RC beams. The major difficulty was NFRP rupture and the debonding failure, as mentioned in another study mentioned in this review. However, research comparing the behavior of unreinforced beams and beams with CFRP and NFRP test results showed that NFRP increased the load-carrying capacities of the unreinforced beam by up to 40%, which was better than that of CFRP. Finally, the study concluded that NFRP exhibits a behavior similar to that of CFRP and, moreover, that NFRP has a higher cost efficiency.

### 3.3. FRP Installation Methods in Structural Elements and Their Failure Modes

For repairing, strengthening, and retrofitting, FRPs are attractive alternatives for improving the resistance of a structure because it is possible to rehabilitate a structure without the need to rebuild and conserve the original facade [4,6].

In recent years, the use of synthetic fibers has attracted attention because of their resistance to high corrosion and other chemical attacks such as chloride and sulfate [1,5], durability, and resistance to flexural and shear [2]. The most commonly used fibers are (i) carbon (CFRP), (ii) glass (GFRP), (iii) aramid (AFRP), and (iv) basalt (BFRP) [6], which can be used in different methods, such as (i) NSM, (ii) EBR, and (iii) EBROG.

Although the majority of the studies presented in this review have proven to be experimental studies with different types of analysis methods, a comparative analysis of the design guidelines for FRP was performed by Mhanna et al. [14]. The authors demonstrated that the all-design provisions provided, on average, unsafe predictions for the U-wrapper configuration for the shear capacity of strengthened RC beams; in other words, the guidelines overestimated the capacity of the wrapped FRP configuration. This could justify the experimental study of the configuration and schemes of FRP reinforcements.

#### 3.3.1. Mechanical Properties Using the EBR Method

**Figure 9:**
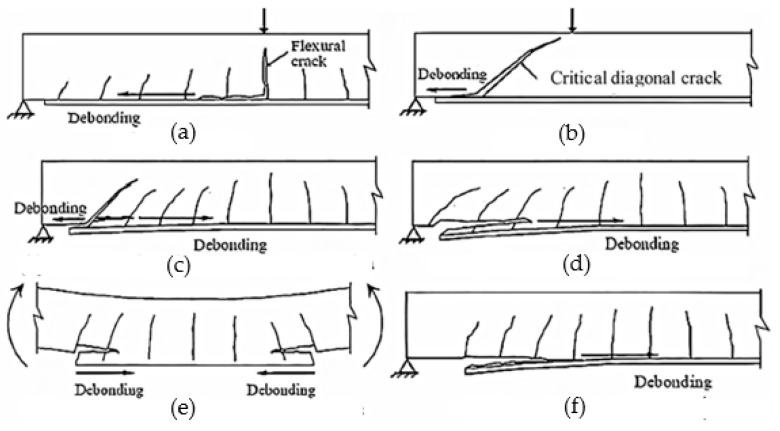
Typical modes of failure of RC beams with FRP using the EBR method [34] (**a**) deonding; (**b**) critical diagonal crack debouding (**c**) critical diagonal crack with concrete cover separation; (**d**) concrete cover separation; (**e**) concrete cover separation under pure bending; (**f**) plate end interfacial debounding.

The EBR is the most commonly used technique for applying FRPs, and its advantage is the simplicity of the construction process [35]. However, most studies indicated five failure modes: (i) FRP rupture, (ii) adhesive failure, (iii) the debonding of adhesive FRP, (iv) the debonding of adhesive concrete, and (v) concrete substrate failure [1]. Therefore, the main issue with the EBR method is the debonding failure (DF) before the ultimate load [15,45], and this type of failure mode is shown in Figure 9.

Saribiyik et al. [15] studied the adhesion performance and the tensile strength capacity of BFRP strips attached to the concrete surface on notched beams employing various anchor types. The study produced a total of 27 notched concrete beams that were tested by four-point flexural testing from two points under constant speed loading to determine the adherence of BFRP strips, which were evaluated by five groups based on the bonding configuration of the BFRP strips. The first group did not have BFRP strips and only evaluated the resistance of the concrete surface by considering the base point; the second group focused on the effective length of the strip; the third group used the technique of the second group but added a U-jacked head; the fourth group combined the techniques of the second and third but added a fan head; and the last group used a steel anchor head with different thicknesses of the BFRP strip.

**Figure 10:**
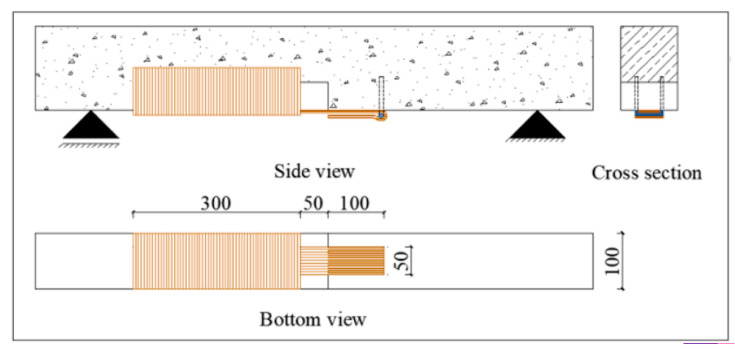
The optimal bonding configuration was reported by Saribiyik et al. [15].

They found that the anchor type significantly affects the load-bearing capacity and bending behavior, as shown in Figure 10; in contrast, the bending length did not.

Most studies indicated that DF recurs and that the stripes separate from the concrete surface. Although the anchored connections delayed the DF and increased the load-bearing capacity, the failure of these specimens was caused by a rupture of the BFRP strip. In conclusion, the specimens that had a steel anchor exhibited a better distribution of tensile stress, resulting in a higher strength of 24–26% more compared with the load-bearing capacity of the group that used a U-jacked head and 29–43% more compared with specimens that used a fan head [15].

Without anchors, Yazdani et al. [46] evaluated the flexural capacity of EBR and found that regular CFRP can improve by 62–78% with DF. However, the authors experimented with a pre-saturated CFRP without any anchor and reported an increase in the flexural capacity of 78%. The anchor increased the flexural capacity by 13%, and the failure mode was delimitation-rupture. However, Yazdani et al. [46] reported a problem similar to that reported by Saribiyik et al. [13].

Hamrat et al. [47], who evaluated the behavior of repaired RC beams, found that FRPs enhance the load-carrying capacity, stiffness, and deflection; however, just two of the seven tested beams failed for FRP sheet rupture; the others showed a delamination or DF of the FRP sheet, under similar conditions as the Yazdani et al. [32] and Hamrat et al. [47] studies.

Similarly, Dong et al. [48] investigated the bond behavior and found that the ultimate load of the bond was enhanced by an average of 52% with a single CFRP anchor. However, Halicka et al. [25] conducted a study on pretensioned CFRP, and the results showed that active pretensioned strips reduce the deflection of timber beams by approximately 26.7% at 60 kN, although Saribiyik et al. [13] concluded that U-jacked was the best configuration for avoiding a DF. Dong et al. [34] affirmed that a DF not only affects the configuration of FRP but also affects the epoxy properties and quality of the concrete surface, which controls the flexural strength of the beams [22,49].

One solution that scholars have presented to reduce DF is to add materials that could improve the bonding of FRP concrete. Liu et al. [15] investigated another FRP strip disposition and proposed the use of CFRP plates with engineering cementitious composites (ECC), by applying EBR with different positions of CFRP plates to strengthen RC beams to compare the strengthening efficiency of RC beams, CFRP-strengthened RC beams, and CFRP-ECC composite-strengthened RC beams.

When one of the CFRP-strengthened RC beams was tested, and the load reached approximately 80% of the ultimate bearing capacity, the CFRP plate was deboned from the surface of the beam; consequently, the beam lost its reinforcement. Similarly, DF has been observed in other studies. In another study, the authors found that the CFRP plate improved the flexural bearing capacity and that the use of ECC reduced delamination, resulting in separation between the concrete surface and CFRP plate, as shown in Figure 10.

The results demonstrated that the use of CFRP can improve the stiffness and ductility [48,50]; however, the load capacity was similar to that of reinforced RC, owing to adhesive failure or delamination, which is similar to the results of other studies. However, Liu et al. [35] demonstrated that the use of ECC as an adhesive layer provides better behavior and reduces beam deflection.

**Figure 11:**
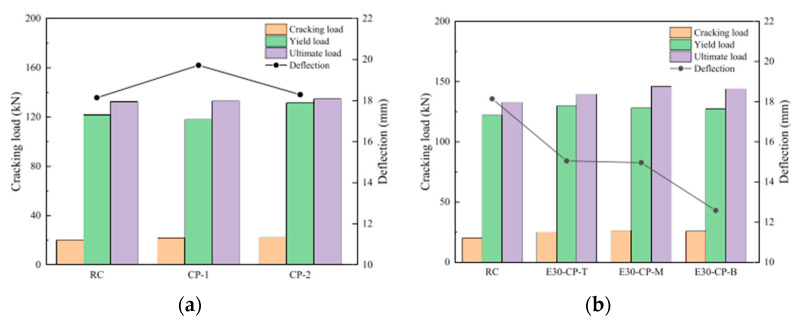
Load–midspan deflection relationship of beams from Liu D. et al. [35]: (**a**) RC beams and RC reinforced with CFRP beams; (**b**) reinforced with CFRP-ECC beams.

Additionally, the location of the CFRP plate can improve the load capacity, as shown in Figure 11, for specimen E30-CP-M, where the CFRP plate is in the middle, and increase the ultimate load with a good deflection.

The Liu et al. [15] study demonstrated that bending shear cracks can cause stress concentrations at the interface, which leads to the debonding damage of CFRP. Xue et al. [51] included shape memory alloys (SMA) when prestressing RC beams and externally bonded them with fiber-reinforced polymer (FRP) sheets. The addition of SMA reduced the formation of diagonal cracks in the RC beams; the shear strength increased by 56.4% with SMA/CFRP and 33.1% with SMA/BFRP, compared to the control beam.

**Figure 12:**
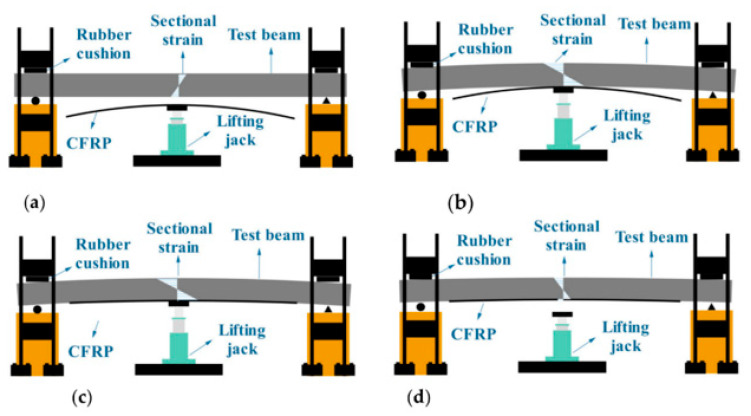
Reverse arch method. (**a**) prepare to apply anti-arching force; (**b**) apply anti-archingforce; (**c**) paste carbon fiber sheet; (**d**) revocation of anti-arch equipment.

Another method for reducing the effects of DF is the reverse arch method. This method was applied by Yu et al. [52] and is shown in Figure 12. They found that, compared to the base beam, the cracking load resistance and ultimate load with the reverse arch method increased by 56% and 63%, respectively. In addition, this method can enhance the rigidity of the beam and reduce the development of cracks, consequently reducing the effects of the DF.

In each study, it was found that the DF and orientation of the fibers could enhance the shear strength and reduce the DF, as proven by Bouyahyaoui et al. [53]. Their investigation demonstrated the effects of the position of FRP strips on masonry walls for applications in historical buildings. They found that using FRP in a diagonal arrangement has better behavior than using full surface reinforcement, and Saribiyik et al. [15] found that, to avoid debonding failure, the laminate must be anchored for a sufficiently long time.

**Figure 13:**
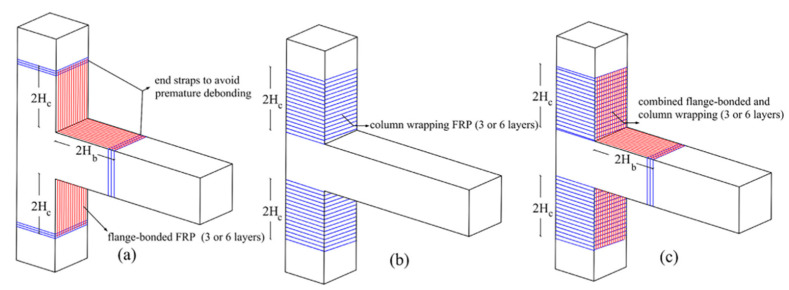
Three different retrofitting schemes. (**a**) Flange-bonded FRP with end straps; (**b**) FRP on columns; (**c**) combined (**a**,**b**).

Configurations and combinations of FRPs could improve the EBR method. Mahdavipour et al. [31] investigated the application of FRPs with different configurations on an ordinary RC building to examine the collapse capacity, displacement ductility, and failure mode. CFRP composites were applied near the beam–column connections and regions prone to plastic deformation using the EBR method, as shown in. To enhance the flexural capacities of beams and columns, the configuration depends on this objective. The first configuration was assumed to be the length of the retrofit. The second scheme was used to improve the ductility of the columns, and the third scheme was a combination of the other two schemes, as shown in Figure 13.

The best capacity results were obtained by the third scheme: it showed a 20% increment in frame capacity, which was equal to that achieved using six layers of flange-bonded FRP. Using a combination of flange-bonded and wrapping layers, with three and six layers, increases the flexural capacity by 20% and 31%, respectively, relative to that of the original frame. In addition, this scheme had a lower collapse probability under the maximum considered earthquake.

Moreover, the second scheme, using three and six layers, demonstrated higher ductility values of approximately 27% and 44%, respectively. Finally, the results demonstrated that variation in the configuration has severe effects on the failure mode of the frame.

A summary of investigations using different types of fibers in FRP, using the EBR method to compare the results, is shown in Table 2.

#### 3.3.2. Mechanical Properties Using the EBROG Method

EBROG can be considered as a modification of the EBR technique. Moghaddas et al. [54] compared the behavior of EBR and EBROG in beams and found that the EBROG specimens presented enhancements of 18–68% in their bond strength compared to the EBR specimens. Consequently, the slip for the maximum on-groove shear stress increased by approximately 50% compared with that recorded for the EBR specimens [55].

As the EBROG method depends on the grooves, Zholfaghari et al. [56] experimented with grooves of different sizes to determine the optimal groove dimensions and validate the related relationships presented in previous studies. The results for specimens with the same cross-section but different large and deep grooves showed that a specimen with a larger groover had a higher load-bearing capacity than that with a larger depth.

The values presented in Table 3 are the results obtained by Zholfaghari et al. [56], who confirmed that increasing the depth beyond these values can decrease the load-bearing capacity of the bond.

The EBROG method can also be used with prestressed FRP, as demonstrated by Moshiri et al. [55]. The results indicated an enhancement in the maximum strain by 12.4% and the slab flexural capacity by 77% compared with the non-prestressed EBR method, because EBROG can transfer the prestress force from FRP to the concrete slab [55]. Zholfaghari et al. [56] reported that the bond strength of the EBROG method was 14.7–73.5% higher than that of the EBR method [56].

Although NSM, EBR, and EBROG are the techniques most used for reinforcement, another technique is called externally bonded reinforcement in grooves (EBRIG). This technique involves introducing a sheet of FRP into the groove so that it is not exposed to the outside, as in the EBROG method.

Zamani et al. [57,58] demonstrated that grooving methods are superior to the EBR method in terms of bonding [54]. EBRIG with one layer of FRP achieved a maximum load 82% and 41% higher than the EBR and EBROG techniques, respectively. Moreover, EBR and EBROG failed to debond, whereas EBRIG failed because of the rupture of the CFRP sheet. The authors affirmed that the reason could be that the CFRP sheet was directly attached to the lateral sides of the groove and confined between the concrete and filler material.

The main problem with the EBR technique is the debonding failure. Some structures have already implemented FRP-bonded systems, because they are prone to debonding failure. Qui et al. [57,58] showed a solution to detect the defect of FRP-bonded structures. The acoustic laser technique is a type of non-destructive testing method that uses mechanical and electromagnetic waves for structural identification, vibration measurement, and other characterization purposes applied at a real construction site.

The authors affirmed that the ability of the test is proven in the laboratory; however, proving the technique in the field is more difficult. Environmental vehicle noise, which can disturb the results and reduce their effectiveness, is the main problem with this method. Although the test method is a good solution for detecting bonded difficulties, in the field, it is necessary to reduce the noise or apply it under different conditions.

#### 3.3.3. NSM Method

Although the EBR technique is most commonly used in the rehabilitation and repair of structures, its main disadvantage is the DF. To enhance the bonding between FRP and concrete, the NSM technique was developed for repairing RC structure systems and masonry structures. However, the failure of NSM is mainly caused by the peeling off of the concrete cover at the end of the FRP bars [59].

Gong et al. [59] studied the use of CFRP bars in the hogging moment regions of a steel–concrete structural system by using the NSM technique. The effects of the anchorage method, failure mode, load-carrying capacity, deflection, strain of CFRP, effective number of bars, spacing of the grooves, and length and cross-sectional area of CFRP were evaluated.

The results showed that the premature debonding of CFRP bars did not occur with or without an anchoring system, indicating that the use of the NSM technique, such as the EBR technique, demonstrates that the failure mode is the buckling of steel and not debonding. Additionally, the length of the CFRP bars affected the load-carrying capacity, and the number of bars increased the ultimate load-carrying capacity, with two and four CFRP bars increasing it by 10% and 20%, respectively. However, the ductility decreased by 14.8% and 19.35%, respectively [59].

GFRP bars are commonly used in the NSM method and present good results, though less so than CFRP bars. Barris et al. [60] studied the flexural behavior of RC beams, and their results reported that the NSM method increases the flexural resistance of beams. Moreover, beams reinforced with GFRP bars required a higher concrete strength because failure was observed for concrete crushing.

The bond behavior between concrete and FRP bars was investigated to prevent mode failure. For example, Gao et al. [61] analyzed the bond–slip behavior between seawater sea sand concrete and CFRP bars with different surface shapes using a pull-off test. The results indicated that the ribbed bar has a significantly higher bond strength than the regular bar. When the ratio of the cover depth to the bar diameter was greater than 4, the CFRP bar failed; otherwise, a concrete splitting failure was observed.

Zhou et al. [62] presented a similar investigation; however, advanced sustainable concrete was used. The authors reported that the ratio of the cover depth to the bar diameter critical value is between 3.5 and 4.5 due to the splitting failure.

A series of anchorage techniques have been developed to prevent a DF using the NSM method. Diab et al. [63] proposed a nonmechanical anchorage technique for shear strengthening using NSM-BFRP bars applied in T-beams. The technique consisted of installing U-shaped hybrid BFRP stirrups. The results showed that the beams without anchorage failed to exhibit the DF of the BFRP bars, and the shear capacity increased by 8.3–46% compared to beams with NSM without anchorage; however, an increasing number of beams with NSM with anchorage, 39.6–81.6%, was proposed

The prestressed strip used in the NSM method could be a solution for a DF. Su et al. [25] proposed a novel method for prestressed CFRP bars, which consisted of prestressing the central area of the strips. However, end cover separation was the dominant failure mode, concrete crushing occurred when the prestress force was quite small, and the strip was sufficiently long because the bond length influenced the flexural behavior. This method increased the bearing capacity by 19% compared to that of the non-prestressed beam.

#### 3.3.4. Scanning Electron Microscopy (SEM) Images and X-ray Diffraction Pattern Results

The flexural strength of the beams is controlled by the epoxy characteristics and the quality of the concrete surface, both of which are influenced by DF [46].

Ramezani et al. [64] mentioned that the impact of varying carbon nanotube-reinforced cementitious material lengths, diameters, and concentrations on the mechanical properties revealed that every size has distinct features and mechanisms influencing the properties of concrete structures; for instance, small fiber diameters increase compressive strength but have a negative effect on flexural strength.

The distribution and orientation of fibers is an important characterization for defining the resistance of an FRP, its epoxy, and the adherence between the FRP–concrete surface. Adekomaya et al. [65] investigated the characterization and morphological properties of glass-fiber-reinforced epoxy composites that were fabricated using different hand lay-up techniques. This study aimed to analyze the influence of varying lay-up approaches on the final properties of composite materials.

The results showed that the hand lay-up technique significantly influenced the mechanical and morphological properties of GFRP epoxy composites. The samples fabricated with the triple-layer lay-up exhibited superior mechanical properties compared to those of the single- and double-layer samples. This can be attributed to the better fiber distribution and interfacial bonding between the layers. The SEM images confirm the presence of well-distributed fibers throughout the composite matrix, further validating the superior performance of the triple-layer samples.

By adjusting the hand lay-up techniques, the impact of the fiber distribution on the composite properties was investigated. A uniform fiber distribution led to improved mechanical properties, whereas an uneven distribution negatively affected the performance of the composites. It was observed that proper handling and control during the hand lay-up process were crucial for achieving an optimal fiber distribution.

The influence of fiber orientation, fiber volume fraction, and interfacial bonding on the mechanical behavior of the composites was thoroughly analyzed by Yu et al. [66]. This study focused on investigating the tensile and flexural behaviors of additively manufactured continuous CFRP. Various tests were conducted to evaluate the tensile and flexural properties of the composites. The experimental results showed that the addition of continuous carbon fibers greatly improves the mechanical strength and stiffness of the composites compared to those of non-reinforced polymer materials. The tensile strength and modulus significantly increased, indicating enhanced resistance to elongation and deformation. Similarly, the flexural strength and modulus also exhibited remarkable improvements.

These spacings and voids had a significant impact on predicting the elastic properties, as well as the tensile and flexural behaviors, of CCFRP specimens. In addition, the strength and ductility were dependent on the carbon fiber concentration. Specimens with a higher carbon fiber concentration exhibited higher strength and lower ductility [66].

SEM analysis is not only used for the orientation or concentration of fibers; Pan et al.’s [67] study employed SEM as a valuable tool to analyze the changes in the microstructure and surface morphology of GFRP and HFRP bars after aging in an alkaline environment. The SEM analysis revealed significant differences between the GFRP and HFRP bars in terms of their response to alkaline aging.

The GFRP bars exhibited minor surface degradation and minimal fiber–matrix debonding, indicating excellent resistance to the alkaline solution. In contrast, the HFRP bars displayed more pronounced microstructural changes, including fiber pull-out, fiber fractures, and matrix cracking, suggesting slightly reduced durability compared to the GFRP bars [67].

Owing to the hybrid composition of the HFRP bars, which combined glass and carbon fibers, microstructural alterations were detected. In an alkaline environment, carbon fibers are more prone to degradation than glass fibers because of their poorer alkaline resistance. Nevertheless, glass fibers add to the overall robustness. Optimizing the composition and creating more robust FRP-reinforcing materials require an understanding of these degradation mechanisms [67].

### 3.4. FRPs in Civil Infrastructure Systems

In the civil industry, FRPs can also be used in new constructions or composite FRP–concrete systems [4,5,6]. Zou et al. [16] created an FRP truss plate joint integrated with a U-shaped gusset plate to compare the behavior of the FRP joint, its failure mode, its load capacity, and its stiffness with that of a traditional join. The results showed that the load-carrying capacity improved by 63% and 42% with conventional bolted and modified bolted joints, respectively. Moreover, the deflection was reduced by up to 33%.

**Figure 14:**
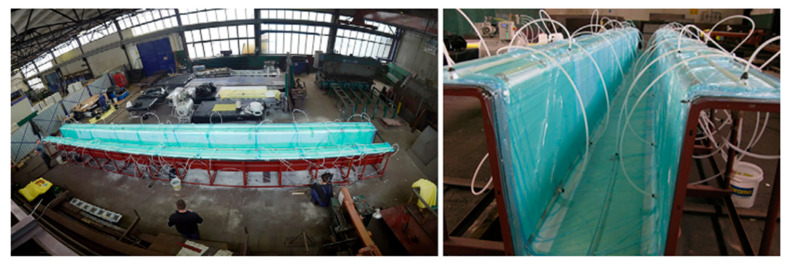
Prototype FRP bridge girder [3].

Since the early 1980s, FRPs have been used to construct large-scale structures for deck systems, foot bridges, and vehicle bridges, particularly in Europe. Siwowski et al. [3] designed, manufactured, and tested an all-composite FRP bridge girder, as shown in Figure 14. Their research consisted of designing and describing the manufacturing process of a prototype girder as part of a modular footbridge and evaluating its structural behavior (the stiffness and strength of the final girder).

The girder was made with glass fiber as a basic reinforcement, carbon fiber for the highly loaded zones, epoxy resin as a matrix of FRP composites, and PVC foam in the case of a sandwich laminate.

Moreover, the girder design was based on the new European guidelines, finite element analysis, and recommendations for existing FRP footbridges. The test indicated three failures: (i) debonding and delamination of the upper flange in the assembly joint, (ii) shear buckling of the web, and (iii) cracking and debonding in the assembly joint of the deck panels. The authors affirmed that some of the failure modes were unexpected and that the girder behaved according to the design. However, the test was unique and, therefore, cannot be statistically verified.

Nevertheless, the authors presented some bridges on which they constructed their own prototypes. The two most commonly used technologies are vacuum-assisted resin transfer molding (VARTM) and pultrusion. Worldwide, bridges have been constructed using these technologies, such as the footbridge “Ooypoort”, located in Nijmegen, The Netherlands, which has the largest span length in the world (approximately 56 m) and is composed of solid laminates and sandwich plates.

## 4. Future Development

From various experimental study cases and full-scale applications of FRPs, it is evident that the future development of FRP will focus on three areas: the reinforcement, rehabilitation, and reparation of structures.

FRP has superior mechanical properties compared to traditional reinforcement methods, such as steel bars. It exhibits better ductility, shear strength, and corrosion resistance. FRPs can replace steel bars in reinforcements. However, the design codes for applying FRP have overestimated their capacities; thus, FRPs have been empirically and experimentally applied in the field. Although many investigations have found that the use of FRP enhances the properties of structural elements in RC, some scholars have recommended investigating the effectiveness of FRP outside of the laboratory, as environmental conditions can change its behavior.

Various fibers in the market for structural reinforcement have enabled cost–benefit solutions, as well as different responses to several objectives, to be obtained. For instance, AFRPs have demonstrated better resistance to marine environments than others, GFRPs have shown a better cost–benefit, and CFRP has shown the best mechanical properties of all currently available fibers but has a higher carbon emission factor than BFRP.

NFRP is a novel method for incorporating environmental care and the principles of a circular economy. However, NFRP does not have the same mechanical properties as synthetic fibers. The use of combined fibers could reduce the manufacturing cost and result in an obvious improvement in the mechanical properties of FRP.

Similarly, methods for applying FRPs have been extensively studied. The future of methods could continue the development of methods to prevent a DF for the EBR method, which has been demonstrated to be effective and the easiest method to apply. Moreover, other materials, such as high-strength mortar, cementitious materials, and chemical products, can be used in the adhesive layer to prevent a DF.

Scholars have proposed different techniques to anchor FRP strips; however, it is essential to apply them to full-scale structures to prove their effectiveness. However, NSM has demonstrated that it is the best reinforcement method; it has shown the difficulty of applying it, so constructors prefer to apply the EBR method. It is necessary to investigate cases that require the application of the NSM method.

EBROG is a promising method that has demonstrated better failure modes and shear strength resistance than EBR. EBRIG is a modified EBROG method that has been proved to have a lower DF than the EBROG and EBR methods. However, this method has not yet been studied.

Studies on FRPs under seismic effects have shown good acceptability between the structure and FRP; however, it is necessary to investigate the failure modes and improvements that could prevent these failures. Variations in the structural parameters, configuration, and installation location of FRPs are schemes that can produce essential changes and results.

## 5. Conclusions

This paper reviewed experimental cases and applications in civil structure systems. The results indicated that the implementation of FRP to reinforce, repair, and rehabilitate structures enhances the shear strength, flexural strength, and load displacement. However, the failure modes were major problems that need to be solved in future investigations.

Fibers Used in FRPs

1. The large number of fibers used for structural reinforcement has enabled various cost–benefit solutions, as well as different responses to several objectives, to be obtained. AFRPs have demonstrated better resistance to marine environments than GFRP or BFRP, which have shown better cost–benefits, and CFRP has the best mechanical properties of all currently available fibers; however, it has a higher carbon emission factor than the others. A solution to the problem of environmental damage from manufacturing synthetic fibers could be the use of NFRP. Although they do not exhibit the same improvements as synthetic fibers, using mixed fibers is an alternative to achieve mechanical requirements in reinforced concrete structures.
2. For repairing/reinforcement structures, CFRP is the most common and effective material because it has a higher tensile strength and modulus of elasticity than other fibers. In addition, some studies have found that CFRP is more resistant to harsh environments than other materials.
3. Under SEM analysis, the impact of the fiber orientation on the composite properties was assessed using various hand lay-up techniques. Composites with the desired fiber alignment displayed superior mechanical performance compared to those with a random fiber orientation. Moreover, a unidirectional fiber alignment resulted in the highest tensile and flexural strengths.

Improvement in the Mechanical Properties

4. Many investigations aimed at studying the mechanical properties affirmed that FRPs have a high modulus of elasticity, high tensile strength, good fatigue resistance, high dimensional stability, low coefficient of thermal expansion, and low abrasion. Most investigations concluded that the flexural and shear capacities could improve by 20–80% and 14–20%, respectively. These percentages could increase with a better configuration of FRP as a U-jacket or by including anchors, which could enhance the epoxy properties and the quality of the concrete surface.
5. The combination of types of FRPs according to the fiber, such as GFRP-CFRP or AFRP-GFRP, could increase the flexural capacity of a structural element by more than 100% and could enhance the load capacity, consequently increasing the elastic modulus between 10% and 14%.

Different Types of Installation

6. The EBR method has five failure modes: (i) FRP rupture, (ii) adhesive failure, (iii) debonding of the adhesive FRP, (iv) debonding of the adhesive concrete, and (v) concrete substrate failure. The main issue with the EBR method is the debonding failure before the ultimate load. Many studies have concluded that anchoring the slips/sheet of FRP applied in the EBR method improves the failure mode and increases the flexure and shear strength. However, the NMS method could be considered as the best and safest method, in terms of failure, and constructors prefer EBR due to the easiness of the install.
7. The FRP jacket can change the mode of failure from brittle shear failure to bending shear failure and can also enhance the ductility capacity of columns; consequently, this novel method can reduce the effects of seismic loads. Similarly, FRPs improve the mechanical properties of damaged structural elements for corrosion, wet–dry cycles, fire, and chemical substances.

## Figures and Tables

**Table 1:** Paper selection parameters.

Parameter of Selection	Description
Publication year	Between 2019 and 2023.
Proposal of investigation	The aim of the investigation must be to quantify a scale and compare it with a specimen “cero” or base.
Method of obtaining the results	The methodology must be described on paper, and the specimens that were evaluated must be tested under a certificate test with calibrated equipment.

**Table 2:** Experimental results using the FRP/EBR method.

Author	Structural Element Tested	Type of FRP Used	Results Compared to a Control Element	Results Compared to Other Fibers
Wang et al. [28]	FRP–concrete surface	CFRP	Under WDC 0–360 days Debonding loads ↓ (less) 29.3% Bond shear stress ↓ 15.5%	Elastic modules = 231 GPa Tensile strength = 4132 MPa
		BFRP	Debonding loads ↓ 38.3% Bond shear stress ↓ 39.7%; T = 4132	Elastic modules = 91 GPa Tensile strength = 210 MPa
Yu et al. [1]	FRP–concrete surface	AFRP	Under WDC 0–300 days Bond shear stress ↓ 14.8–20% Bonding resistance ↓ 22.6–34.2%	-
Toufigh et al. [27]	Tensile lap	BFRP	Under acidic environments Bond strength ↓ 18%	-
		CFRP	Bond strength ↓ <10%	
		AFRP	Bond strength ↓ <10%	
Sreekumar Kavitha et al. [5]	Concrete columns	CFRP	Axial load carrying capacity ↑ 57%	-
		GFRP	Axial load carrying capacity ↑ 23%	
Shi et al. [44]	RC columns	LRS FRP (PET FRP)	Under high axial load Ductility coefficient ↑ (more) 190–310.1% Cumulative energy dissipation ↑ 2342.6–5660.4%	Under low axial load Ductility coefficient ↑ 300% compared to CFRP Cumulative energy dissipation ↑ 1962.8% compared to CFRP
Kalyani et al. [23]	RC beams	CFRP-GFRP (hybrid FRP)	Strength ↑ 81% Flexural capacity ↑ 102.63–202.63%	-

**Table 3:** The relationship between the effective groove width/depth (Zholfaghari et al. [56]).

Groove Depth (mm)	Effective Groove Width (mm)
2.5	5
5	10
10	5
15	5

## Data Availability

Not applicable.
